# Delayed Graft Function in Kidney Transplant: Risk Factors, Consequences and Prevention Strategies

**DOI:** 10.3390/jpm12101557

**Published:** 2022-09-21

**Authors:** Claudio Ponticelli, Francesco Reggiani, Gabriella Moroni

**Affiliations:** 1 Independent Investigator, via Ampère 126, 20131 Milano, Italy; 2 Nephrology and Dialysis Unit, IRCCS Humanitas Research Hospital, Via Manzoni 56, 20089 Milan, Italy; 3 Department of Biomedical Sciences, Humanitas University, 20090 Milan, Italy

**Keywords:** delayed graft function, acute kidney injury, ischemia–reperfusion injury, maladaptive repair, mitochondrial dysfunction, rejection

## Abstract

Background. Delayed graft function is a frequent complication of kidney transplantation that requires dialysis in the first week posttransplant. Materials and Methods. We searched for the most relevant articles in the National Institutes of Health library of medicine, as well as in transplantation, pharmacologic, and nephrological journals. Results. The main factors that may influence the development of delayed graft function (DGF) are ischemia–reperfusion injury, the source and the quality of the donated kidney, and the clinical management of the recipient. The pathophysiology of ischemia–reperfusion injury is complex and involves kidney hypoxia related to the duration of warm and cold ischemia, as well as the harmful effects of blood reperfusion on tubular epithelial cells and endothelial cells. Ischemia–reperfusion injury is more frequent and severe in kidneys from deceased donors than in those from living donors. Of great importance is the quality and function of the donated kidney. Kidneys from living donors and those with normal function can provide better results. In the peri-operative management of the recipient, great attention should be paid to hemodynamic stability and blood pressure; nephrotoxic medicaments should be avoided. Over time, patients with DGF may present lower graft function and survival compared to transplant recipients without DGF. Maladaptation repair, mitochondrial dysfunction, and acute rejection may explain the worse long-term outcome in patients with DGF. Many different strategies meant to prevent DGF have been evaluated, but only prolonged perfusion of dopamine and hypothermic machine perfusion have proven to be of some benefit. Whenever possible, a preemptive transplant from living donor should be preferred.

## 1. Background

The definition of delayed graft function (DGF) varies among transplant centers, although most define it as an acute kidney injury (AKI) that occurs in the first week after transplantation and requires a dialytic treatment [1,2,3]. DGF is a frequent complication, with rates ranging between 25% and 30% [4], and is still a major clinical challenge for the management of kidney transplant recipients. In fact, DGF is associated with higher rejection rates and worse short- and long-term outcomes [5].

This minireview will be focused on the risk factors, consequences, and possible prevention of DGF. Causes of early acute graft failure, including vein or artery thrombosis, non-viable kidney, ureteral obstruction, and hyperacute rejection will not be taken into consideration.

## 2. Risk Factors for DGF

Several factors may influence the development of DGF (Table 1).

The major risk factors involved in the development of DGF are ischemia–reperfusion injury (IRI), the source of donated kidney (deceased vs. living donation), the quality of the donated kidney, and the clinical conditions of the recipient.

### 2.1. Ischemia–Reperfusion Injury

The removal of the kidney for transplantation involves a warm ischemia time (WIT) and a cold ischemia time (CIT), followed by blood reperfusion after transplantation. WIT, which is defined as the period between arterial clamping and the start of the infusion of cold fluid, is usually short and has little impact on early kidney function in transplants from living donors or brain-dead donors. However, in the case of a kidney with multiple arteries or donation after circulatory death, WIT may be longer. A WIT duration longer than 45 min represents an increased risk for DGF and graft failure [6,7]. Additionally, the length of CIT, which is defined as the time in which the organ is preserved in a hypothermic state before proceeding to transplantation, may influence the outcomes of kidney transplants. In transplants from donors after circulatory death, a CIT shorter than 24 h is usually accepted. However, better outcomes may be reached with a shorter CIT. In fact, it has been reported that an additional hour of CIT may increase the risk of graft failure [8]. The deleterious effects of prolonged CIT are enhanced in recipients of kidneys from older donors [9,10]. The increasing acceptance of older and higher risk donor kidneys has reduced enthusiasm for simple cold storage, which was previously made possible by the development of better organ preservation solutions [11]. Therefore, there has been a shift back to continuous machine perfusion, though improved with novel technologies that include oxygenation and modifications of perfusion solutions [11]. Presently, it has been demonstrated by a recent metanalysis that the use of hypothermic machine perfusion (HMP), rather than traditional static cold storage, reduces the risk of DGF [12].

**Figure 1:**
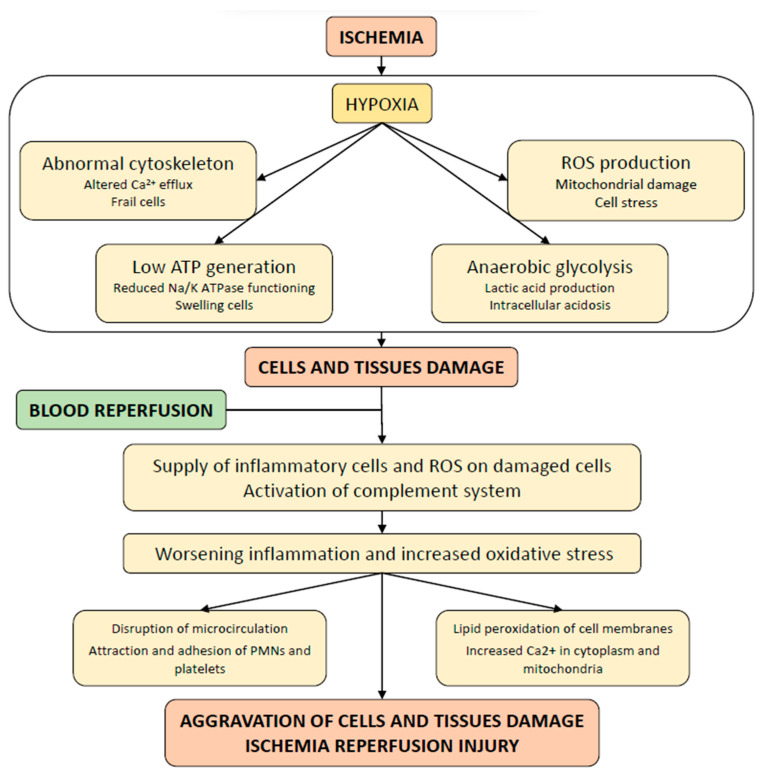
Warm and cold ischemia may produce hypoxia of the kidney tissue.

Before discussing the mechanistic interpretation of IRI (Figure 1), it should be reiterated that in case of a donor with brain damage, renal ischemia due to systemic vasoconstriction of the donor may be present even before kidney removal.

Epithelial tubular cells and endothelial cells are particularly sensitive to ischemia and their damage is proportional to the duration of the ischemia. Hypoxic cells cannot produce an adequate quantity of ATP. The insufficient activity of the ATPase pump creates an imbalance of sodium/potassium exchange with intracellular accumulation of sodium ions. The cells become overloaded and swell. Simultaneously, the anaerobic glycolysis generates lactate that results in intracellular lactic acidosis. Hypoxia and defective production of ATP lead to cytoskeletal abnormalities and impaired mitochondrial efflux of calcium ions promoting superoxide generation, further mitochondrial dysfunction, and cell stress. The combination of these deleterious events may eventually lead to cell death and tissue damage. After reperfusion, the ischemic kidney receives reactive oxygen species and inflammatory cells that, paradoxically, can lead to further cell and tissue damage. The response to inflammation includes impaired microcirculation with vasomotor dysfunction, reduced capillary perfusion, adhesion of leukocytes and platelets with activation of the coagulation cascade. Oxygen stress includes peroxidation of cell membranes and increased influx of calcium ions leading to mitochondrial dysfunction.

During prolonged CIT, the hypoxic cells try to maintain ATP levels through an increased glycolysis induced by the hypoxia inducible factor-1α (HIF-1α) [13]. Despite the high rate of anaerobic glycolysis, ATP levels decrease, while lactic acid, the ultimate product of glycolysis, increases, leading to intracellular acidosis. Due to the shortage of ATP, the activity of the sodium/potassium ATPase pump is deranged, which can disrupt the equilibrium between intracellular potassium ions and extracellular sodium ions. Cells swell due to this osmotic imbalance and start to express adhesion molecules, which promote leukocytes infiltration of the graft. The anaerobic metabolism generates only a minimal amount of high-energy phosphates, which are not sufficient to meet the demands of aerobic tissue. Therefore, active Ca^2+^ efflux is reduced, the cytoskeleton is disorganized, and the overloaded cells appear swollen and frail, and eventually may become necrotic [14,15]. After surgical anastomosis with the recipient circulation, the transplanted kidney, which is already damaged by ischemia, receives oxygen and inflammatory cells that, paradoxically, can cause further damage or worsen pre-existing injuries through oxidative stress production and local inflammation [16]. Complement activation may further aggravate the kidney damage. Animal studies suggested that complement might be activated by lectin pathway [17,18] or by alternative pathway [19]. Complement activation not only can induce an inflammatory response, but may also favor the development of adaptive immunity and trigger the coagulation pathways [20]. Proximal tubular cells and endothelial cells are particularly sensitive to these harmful effects of blood reperfusion [21,22].

The low concentration of anti-oxidative agents in ischemic cells favors the production of reactive oxygen species (ROS), generated by multiple enzymatic sources [23]. ROS activate metabolic, inflammatory and immune processes, eventually leading to cell damage or death [16]. Proinflammatory cells and cytokines may induce loss of tubular cells by apoptosis or epithelial-to-mesenchymal transition, which ultimately leads to interstitial fibrosis [24,25,26]. A large production of ROS can decrease nitric oxide synthesis and contribute to the initiation and progression of endothelial dysfunction [27]. Moreover, inflammatory processes can activate injured endothelial cells and cause their dysfunction, leading to upregulation of coagulation factors and thrombosis [28].

### 2.2. Source of Donated Kidney

Kidneys for transplantation may be removed from living or deceased donors. Living donors may be divided into relatives of the recipients (parents, grandparents, siblings, sons) or unrelated donors (spouses, friends, or Samaritan individuals). Deceased donors may be brain-dead donors (with irreversible brain injury and persistent circulation maintained by supportive measures) or donors after circulatory death. The risk of DGF is higher for deceased donors [29,30,31,32], although it is also present in living donors [33]. Several factors may explain the better outcomes of living-donor transplant (Table 2).

Living donors are generally healthy individuals, or those with only minor comorbidities, and with normal renal function. Current transplant screening tests minimize the risk of transmitting infections or malignancies. Finally, pre-emptive transplantation, which favors better renal outcomes, is easier to realize with living donors compared to deceased donors [34].

Instead, in deceased donors, it may be difficult to obtain a reliable clinical history, and the short available time before transplant may preclude in-depth analyses. Information on clinical history is even more difficult to obtain in case of donors after circulation death. In brain-dead donors, there is severe hemodynamic instability, hypovolemia, and hypotension that can cause kidney hypoperfusion and renal ischemia. Hyperactivity of the sympathetic system and the use of vasoconstrictors to support circulation can further aggravate renal ischemia. In addition, intracranial hypertension causes an enormous production of cytokines and growth factors that promote inflammation and worsen ischemia [35,36].

Brain-dead donors over the age of 60 or those aged 50–59 with two of these abnormalities (history of high blood pressure, final serum creatinine greater than 1.5 mg/dL (133 mmol/L), or cerebrovascular cause of death) are defined as expanded criteria donors (ECDs) [37]. ECDs are often excluded from donation. When kidneys from ECDs are accepted, they often present DGF (see Quality of the kidney section).

A particular problem may be posed by donors with AKI. Despite a high incidence of DGF, graft survival may be obtained, although with inferior outcomes to those observed with standard donors [38,39,40,41,42,43,44,45]. In the cases of successful kidney transplant from donors with AKI, it is possible that pretransplant, AKI was functional, being caused by the systemic and renal vasoconstriction related to the sympathetic overactivity and medications used to sustain circulation. Thus, kidney donors with AKI, and even those with oligo-anuria, may be accepted unless they have relevant comorbidities, are older than 65 years, or have shown evidence of low levels of glomerular filtration rate (GFR) in the past.

Kidney transplant during the present coronavirus disease 2019 (COVID-19) pandemic represents a novel challenge. Large multicenter studies reported that COVID-19 infection was associated with a high risk of acute renal failure and mortality in hospitalized transplant recipients [46,47]. During the pandemic, two different behaviors have been observed in patients on the waiting list: some patients refused kidney transplant for the fear of life-threatening COVID-19 infection, while the others accepted [48,49,50,51]. The reasons remain unknown. Our opinion is that a potential recipient should accept a kidney from a deceased donor, provided that a quick swab resulted negative. However, transplants from living donors may be delayed to periods with reduced risk of contagion. At any rate, according to the last annual report of the US Organ Procurement and Transplantation Network/Scientific Registry for Transplant Recipients (OPTN/SRT), the total number of kidney transplants decreased slightly in 2020. The decrease of total kidney transplants was mainly due to a decrease of living donor transplants [52].

### 2.3. Quality of the Kidney

Donor organ shortage, growing waiting lists, and substantial organ discard rates are key problems for transplantation. The critical importance of organ quality in determining long-term function is becoming increasingly clear. However, organ quality is difficult to predict. Van Moos reviewed the most frequent systems used to assess the kidney quality, including interpretation of renal biopsy, molecular markers associated with biological age, clinical scores, and molecular analyses of peri-transplant biopsies [53]. However, no consensus exists about the clinical validity of these analyses. Other researchers focused on molecular investigations with tools such as epigenetics, transcriptomics, proteomics, and metabolomics [54]. A recent study reported the potential role of pretransplant transcriptomic biomarkers for predicting posttransplant outcome [55]. However, this promising result should be confirmed by larger studies, and the clinical application remains doubtful. While waiting for the possible development and availability of simple and reliable biomarkers, at present, the evaluation of kidney quality rests on the opinion of the clinician. For living donors, there is agreement that patients being considered for donation should be healthy or have only mild diseases that do not cause functional limitations. Most centers use serum creatinine and creatinine clearance to estimate the GFR. In evaluating whether the values of GFR are normal, one should consider that the values of serum creatinine may be influenced by diet, muscle mass, and physical exercise. In particular, the values of GFR change with age. After the age of 30 years, GFR declines by about 8 mL/min/1.73 m^2^ per decade, although with individual variations [56]. Additionally, sclerosis score (defined by total number of sclerotic glomeruli, presence of tubular atrophy, interstitial fibrosis > 5% of renal specimen, and any sclerosis) progressively increases with age [57]. In many centers, a double-checked GFR lower than 80 mL/min/1.73 m^2^ and/or comorbidity leading to potential complications for the donor or recipients represent criteria of exclusion for living donors, with possible exceptions related to particular cases.

More complex is the decision regarding kidneys from deceased donors. There are no major problems for young adults with a GFR greater than 60 mL/min/1.73 m^2^ and an absence of comorbidities. However, many potential donors present lower values of GFR and some of them can even be oliguric. As pointed out above, AKI in the donor should not represent a formal contraindication to the use of those kidneys, but it is important to establish in which setting AKI developed. The age of the donor, the presence or absence of comorbidities, the clinical history, and previous levels of serum creatinine (when available) can help guide acceptance or refusal.

The main issue concerns ECDs. Kidney allografts from ECDs have two-fold increased risk of DGF, more frequent acute rejection, and lower graft function in the long-term [58,59]. However the patient survival was better than that observed in patients on dialysis who remained on the waiting list [60]. Three main strategies may be adopted with kidneys from ECDs: (i) the two kidneys may be assigned to two recipients; (ii) the two kidneys of the donor are given to a single recipient; (iii) the kidneys of old donors are assigned to old recipients. The results with the first strategy were analyzed by a systematic review and meta-analysis on 4833 first transplants. Graft failure was 1.75 times greater in ECDs recipients compared with recipients of standard criteria donors. Using propensity score, the graft failure was 1.34-fold higher in ECDs recipients. With a 10-year follow-up, this difference corresponded to an 8 month decrease of the mean time to graft failure due to ECDs transplantation [61]. In a French study, the outcomes of 170 kidney transplants from marginal donors refused by at least two centers were compared with those of 170 kidney transplants from optimal deceased donors. Delayed graft function occurred more frequently and serum creatinine was higher in marginal kidneys, but the 5-year graft survival (70.4% vs. 76.7%) rates were similar [62]. Some single-center observational studies could not find differences in graft survival rates between transplants from ECDs and transplant from standard-criteria donors [63,64,65].

Several investigators transplanted two kidneys of the same ECDs to a single recipient. Several criteria have been used for choosing this strategy. Some authors opted for a dual kidney transplantation if the terminal creatinine clearance of the donor was lower than 90 mL/min/1.73 m^2^ [66]. Other physicians transplanted two kidneys to a single recipient on the basis of age older than 75 years [67]. Others used histological scores [68]. None of these criteria are free of criticism. Nevertheless, good results with dual transplantation have been outlined by observational studies [69,70,71]. Only small differences or similar patient and graft survival rates were observed between dual and single transplantation when kidneys were allocated according to reliable clinical or histological scores [72,73]. One may wonder if the half-life of a dual transplant is superior to the sum of graft half-lives of two marginal kidneys transplanted in two recipients.

On the 1st of January 1999, the Eurotransplant Senior Program proposed that kidneys from donors aged ≥ 65 years had to be allocated to recipients ≥ 65 years, regardless of human leukocyte antigen compatibility. To reduce prolonged cold ischemia, kidneys had to be transplanted within the European nations participating in the program. In 2010 allocation criteria were modified by listing patients ≥65 years on a separate local waiting list and allocating grafts ≥ 65 years exclusively to older recipients included in the list. Compared to the historical cohort, locally allocated recipients experienced significantly shorter time on dialysis and had better graft survival in the long-term [74]. The success of the program induced a Consensus Meeting to propose that kidney organs from 65- to 74-year-old donors could also be allocated to 55- to 64-year-old recipients [75]. However, the Achilles heel of marginal donors remains the high rate of DGF, independent of the strategy used.

### 2.4. Clinical Management of the Recipient

Most candidates for kidney transplant are patients on chronic dialysis. Patients with hemodynamic instability during hemodialysis or patients submitted to excessive ultrafiltration may develop hypotension that may be aggravated by anesthesia and surgery, eventually exposing them to a higher risk of DGF. These patients may be difficult to manage in the case of urgent dialysis immediately before transplant. Measures to prevent intradialytic hypotension include midodrine hydrochloride, mannitol, albumin infusion, and the use of isothermic or cool dialysis treatment [76,77,78]. If these maneuvers are unsuccessful, it is better to delay transplantation for a few hours to obtain correction of hypotension and hemodynamic stabilization. Antibiotic prophylaxis is probably useless, those centers still using a single shot of antibiotics should prefer cefazolin. At any rate, in the peri-operative period, nephrotoxic antimicrobials, such as aminoglycosides, vancomycin, quinolones, and amphotericin B should be avoided unless strictly necessary. Caution is needed with the use some antalgic agents. Rare cases of interstitial nephritis or acute renal failure have been reported during treatment with nonsteroidal anti-inflammatory drugs or overdose of paracetamol [79,80]. Calcineurin inhibitors (CNIs) are the mainstay of standard immunosuppression for transplant recipients. CNIs are vasoconstrictor agents and may cause an acute increase of serum creatinine. CNIs may also exert direct kidney toxicity that may range from mild tubular changes (isometric vacuolization, giant mitochondria, microcalcification) to arteriolopathy, characterized by focal myocyte necrosis in the media of small arteries in the absence of intimal changes [81]. The most severe form of acute toxicity is represented by thrombotic microangiopathy, with clinical features of hemolytic-uremic syndrome that can lead to graft loss [82,83]. To reduce CNIs exposure and toxicity, several different induction therapies have been used. Some investigators avoided the use of CNIs in the early post-transplant period by replacing CNIs with basiliximab, anti-thymocyte globulins, or belatacept to prevent rejection [84,85,86,87]. A different approach consists in initiating immunosuppression with mammalian target of rapamycin (mTOR) inhibitors associated with low-dose CNIs [88,89]. Most transplant units now initiate immunosuppressive therapy with low doses of tacrolimus associated with basiliximab, mycophenolate, and corticosteroids.

## 3. DGF and Chronic Graft Dysfunction

There is clinical evidence that DGF can induce the development of chronic graft dysfunction [90,91,92,93]. Three main mechanisms may lead to sclerotic lesions: (i) maladaptive repair of lesions caused by AKI; (ii) mitochondrial dysfunction; (iii) activation of rejection mechanisms.

### 3.1. Maladaptive Repair

In DGF, the kidney tends to repair the lesions caused by AKI. However, the response depends on the degree of injury. In mild or subclinical AKI, the injury is followed by inflammatory cells infiltration and tubular cells proliferation. These mechanisms of repair result in an adaptive response with complete resolution of lesions. However, in the case of severe injuries or in the presence of pre-existing renal damage, the response is overwhelmed by injury and fibrosis develops leading to chronic changes [94]. The pivotal process that leads to fibrosis originates from tubular epithelial cells (TECs). In TECs, a severe injury is aggravated by vigorous inflammation leading to arrest of the cell cycle at G2/M phase. At this stage, the intracellular DNA lesions are difficult to repair. During the activation of the DNA repair, TECs adopt a senescent profibrotic phenotype, which also affects other epithelial cells, pericytes and the immune system. There is a strong production of profibrotic factors, which stimulate the proliferation of fibroblasts, the synthesis of extracellular matrix and the epithelial-mesenchymal transition [95,96]. During the epithelial–mesenchymal transition, the TECs progressively lose their polarity and intracellular adhesivity, enhancing their capacity for migration and invasion, and acquire the capacity to transform to mesenchymal cells. Epithelial mesenchymal transition may contribute with tissue injury and inflammation to the development of interstitial fibrosis or may directly cause renal fibrosis [97].

### 3.2. Mitochondrial Dysfunction

Another contributor to chronic renal damage induced by DGF is mitochondrial dysfunction. Mitochondria are the site of ATP synthesis, intracellular calcium homeostasis, and ROS levels, and can regulate the cell cycle control. In DGF, there is an important dysfunction of mitochondria that may induce tubular injury and chronic renal damage [98]. Nephrons are rich in mitochondria that oxidize fatty acids to produce ATP. In damaged tubular cells, fatty acid oxidation is reduced, causing ATP depletion, cell death and dedifferentiation, and intracellular lipid deposition, the same phenotypes observed in fibrosis [99,100]. Injuries to tubular epithelial cells cause cytoskeleton rearrangement and increased production of extracellular matrix. If these lesions do not recover properly, they can participate to mesenchymal reprograming of renal epithelial cells, increasing the risk of developing chronic lesions as a consequence of AKI [101].

### 3.3. Acute Rejection

Fragments of cells damaged or killed during DGF are recognized as danger-associated molecular patterns (DAMPs) by the toll-like receptors and other pattern-recognition receptors. These receptors recruit adaptor proteins to activate a cascade of kinases that amplify and transmit the signal to transcription factors such as nuclear factor kappa-light-chain-enhancer of activated B cells (NF-κB), activator protein-1 and interferon type III. These factors induce pro-inflammatory genes to produce inflammatory cells and molecules of the innate immunity [102]. In the inflammatory milieu, tolerogenic dendritic cells become mature, intercept the alloantigen and migrate to lymph nodes where they present the antigen to quiescent T cells. Through an additional co-stimulating signal, T cells are activated and stimulated by cytokines to proliferate and differentiate into Th1 and Th17 effector cells that can trigger a T-cell-mediated rejection. The collaboration between T and B cells may also favor the production of plasmacytes and antibodies with production of antibody-mediated rejection [103,104]. In summary, there is a complex interplay of various allorecognition mechanisms, which may include the presence of HLA donor-specific antibodies and non-HLA antibodies [105,106]. The important role of DAMPs and innate immunity may explain the frequent association between DGF and acute rejection [93,105,106,107,108]. Modern immunosuppressive therapy has considerably reduced the incidence and severity of acute rejection, but several papers have outlined the poorer long-term graft survival in transplant recipients with DGF and rejection compared to patients who experienced neither DGF nor rejection [92,109,110,111,112].

## 4. Prevention of DGF

Theoretically, the ideal situation to prevent DGF is a preemptive living transplant from a young adult, with normal kidney function and absence of extra-renal pathology, to a young recipient without extra-renal pathology. However, in the real life, this situation rarely occurs. In transplants at risk of DGF, several preventive measures have been considered, but only a few of them have been used in clinical practice [113]. Reducing WIT and CIT is important. The length of WIT largely depends on the surgeons. CIT may be shortened if donor and recipient are in adjacent areas. However, many centers use HLA compatibility for assigning the kidney, accepting the possibility of greater distances between donor and recipient. The research focused on recipient preconditioning has not generated clinically helpful interventions. Donor pretreatment with dopamine prior to procurement has been reported to lower the risk of DGF [114], but a randomized controlled trial failed to find significant difference in graft survival between recipients given dopamine or placebo. However, the same trial outlined that time of administration is important: an infusion time of 7 h improved early graft function [115]. A potential benefit may be obtained by the use of HMP instead of static cold storage. Ten years ago, a review of seven studies reported that HMP reduced the incidence of DGF and increased one-year graft survival [116]. More recently, a meta-analysis of 15 studies performed in the last decade showed that HMP is superior to static cold storage both in brain-death donors and in donors after circulatory death [12]. Thus, HMP may offer a significant reduction in the rate of DGF, even in the modern era. The cost is elevated, but in comparison with standard cold storage, HMP may obtain a better life expectancy and better quality-adjusted life-years [117].

Given the role of complement in IRI, the components of the complement cascade may be possible therapeutic targets. Several studies on drugs that directly target the complement system (e.g., anti-C5), and on complement inhibitors (e.g., C1, C3, factor B and factor D inhibitors), are in progress. However, no protocols have been already approved and further studies are needed to ascertain the role of these drugs in IRI [20].

## 5. Conclusions

DGF is a frequent complication of kidney transplant. Ischemia–reperfusion injury is a main cause of DGF. Proximal tubular cells and endothelial cells are the privileged victims of IRI and can initiate a chain of events leading to chronic graft dysfunction. Delayed graft function is more severe in kidneys coming from deceased donors with reduced kidney function and/or comorbidities. An appropriate management of the recipient in the peri-operative period can prevent AKI and its consequences. The occurrence of DGF may expose the transplanted kidney to maladaptation, mitochondrial dysfunction, and increased risk of rejection, eventually leading to chronic graft dysfunction and graft loss. The increasing use of marginal donors to face the shortage of kidneys makes urgent the development of measures to reduce the impact of DGF on transplant outcomes.

## Figures and Tables

**Table 1:** Factors associated with DGF development. CIT, cold ischemia time; HLA, human leukocyte antigens; WIT, warm ischemia time.

Donor Related	Recipient Related	Perioperative
Deceased donor	Pretransplant dialysis	Hemodynamic instability
Expanded criteria donor	Previous kidney transplant(s)	Calcineurin inhibitors
Donation after cardiac death	HLA mismatch	Nephrotoxic antibiotics
Longer WIT and/or CIT	ABO incompatibility	Nephrotoxic analgesics
Organ quality	Comorbidities	
Donor age	Higher body mass index	
Acute Kidney Injury	African American ethnicity	
Higher body mass index		
Shipping distance		
African American ethnicity		

**Table 2:** Factors that determine better outcomes in living-donor transplant.

	Living Donor	Deceased Donor
Glomerular filtration rate	Usually, normal	Often subnormal
	(>80 mL/min/1.73 m^2^)	(>50 mL/min/1.73 m^2^)
Hemodynamic status	Normal	Unstable
		Severe hypotension
Pre-transplant kidney ischemia	Absent	Severe
Cold ischemia time	Very short	Often prolonged
Ischemia–reperfusion injury	Mild	Severe
Innate immunity activation	Mild	Strong

## Data Availability

The study did not report any data.
